# A Novel Selection Approach for Genetic Algorithms for Global Optimization of Multimodal Continuous Functions

**DOI:** 10.1155/2019/8640218

**Published:** 2019-12-05

**Authors:** Ehtasham-ul Haq, Ishfaq Ahmad, Abid Hussain, Ibrahim M. Almanjahie

**Affiliations:** ^1^Department of Mathematics and Statistics, International Islamic University, Islamabad, Pakistan; ^2^Department of Mathematics, King Khalid University, 61413 Abha, Saudi Arabia; ^3^Statistical Research and Studies Support Unit, King Khalid University, 61413 Abha, Saudi Arabia; ^4^Department of Statistics, Quaid-i-Azam University, Islamabad, Pakistan

## Abstract

Genetic algorithms (GAs) are stochastic-based heuristic search techniques that incorporate three primary operators: selection, crossover, and mutation. These operators are supportive in obtaining the optimal solution for constrained optimization problems. Each operator has its own benefits, but selection of chromosomes is one of the most essential operators for optimal performance of the algorithms. In this paper, an improved genetic algorithm-based novel selection scheme, i.e., stairwise selection (SWS) is presented to handle the problems of exploration (population diversity) and exploitation (selection pressure). For its global performance, we compared with several other selection schemes by using ten well-known benchmark functions under various dimensions. For a close comparison, we also examined the significance of SWS based on the statistical results. Chi-square goodness of fit test is also used to evaluate the overall performance of the selection process, i.e., mean difference between observed and expected number of offspring. Hence, the overall empirical results along with graphical representation endorse that the SWS outperformed in terms of robustness, stability, and effectiveness other competitors through authentication of performance index (PI).

## 1. Introduction

The basic idea of genetic algorithms (GAs) was originated by John Holland in 1960s and was further developed in his book “Adaptation in Natural and Artificial Systems” published in 1975 [1]. GAs are the most efficient procedure to understand and solve problems for which have limited information. These algorithms are able to effectively handle both unconstrained and constrained optimization problems depending on a process of natural selection through biological evolution. The working mechanism of GAs is linked with a search space that contains all possible solutions. Each part of the search space represents one sufficient solution, and its fitness values will be marked by these sufficient solutions, and a set of these solutions is called a population. A set of sufficient solutions will be carried on to the next generation, but weak solutions will be dead based on “survival of fittest” by Darwin's theory of evolution [2].

There are two significant points in the GA process: one is starting point initialization in search space and other is assigning of fitness function [3]. GA starts with the initialization of a population or potential solutions of the problems. This initialization is represented by the chromosomes (individuals), which are a set of genes, with each gene carrying the features of dataset. These chromosomes have their own fitness values depending on the objectives function, so it is very important to determine the solvable objective function.

GA works with the set of solutions and not the decision variables like the other statistical techniques [4–6]. After creating the solutions which are represented by the chromosomes, each of these chromosomes will be evaluated for their fitness depending on the fitness function. Chromosomes that have the fittest value will survive to the next generation. The fitness function depends on the objective of the problem statement. Most of the fitness will be made equal to the objective function value. If the problem statement is to have a minimum cost of some product, then the optimization function here is to find the lowest of the fitness values [7]. Specification in the fitness function is one of the crucial problems in GA because it will determine which chromosomes can survive to the next generation and which will be eliminated from the population.

During the GA process, the feasible solutions in the search space cannot be obtained without reproduction and recombination. Reproduction phase of GA is initiated by the selection of better individuals that will produce new offspring for the next generation with the hope that the next generation will be improved. The core idea of the selection procedure is to enhance the quality of solutions by giving preference to the most suitable individuals and avoiding bad individuals. By combining the current population's solutions, the new population will hopefully contain better solutions and avoiding loss of genetic material. Furthermore, to make the process more reliable, some of the features in the solutions will be mutated or changed with minor probability. The purpose of crossover and mutation will definitely support to generate better a population than the old one [2, 3].

**Figure 1:**
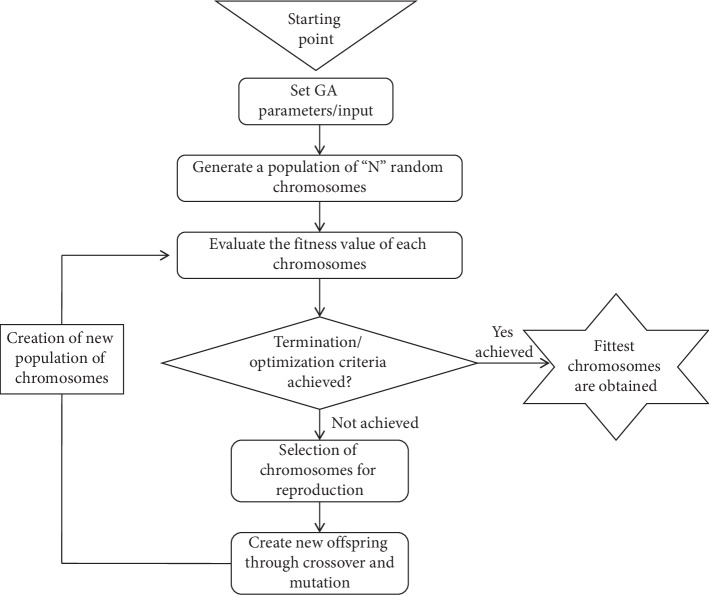
Layout of genetic algorithm.

As we can notice from Figure 1, GA is a stochastic-based heuristic search procedure which is used to set problem-based parameters and making decisions about the following:

- Generate initial population
- The process of parents' selection for reproduction of offspring
- Crossover and mutation of individuals
- Predefine stopping criteria

The function of elitism is to make sure that the good and strong chromosomes can be carried to the next generation by storing them outside the current population. Elitism is helpful in presenting the best solution during the process of crossover and mutation [8, 9]. This can be applied in many ways; one way is to combine both parents and child to produce a new population with all competing to survive to the next generation. The use of elitism can help to converge at a global optimal solution [10].

In order to converge at global optima and avoid the local stagnation, a systematic tradeoff mechanism between exploration and exploitation is compulsory. Most of the stochastic-based heuristic search algorithms try to create a balance between two contradictory measures of their performance: exploration (population diversity) and exploitation (selection pressure). Exploration means the capability of an algorithm to search or explore every region of the possible search space and exploitation means to converge at the optimum solution as soon as possible. The suitable adjustment between exploration and exploitation increases the performance of the GA. In this paper, we will handle this problem with help of the proposed selection procedure. The aim of the selection procedure is to exploit the suitable features of fitted individuals in the context of improved solutions, which technically guide the GA for the convergence to a feasible solution for optimization problem [2]. The GA is widely used in various fields of human endeavor including machine learning [11], scheduling [12], signal processing [13], energy [14], robotics [15], manufacturing [16], mathematics [17], routing [18], and many more.

The rest of the paper is organized as follows: concise detail about some conventional selection schemes is discussed in Section 2. Mathematical derivation along with proposed selection scheme is presented in Section 3. Detailed description about benchmark functions are defined in Section 4, while simulated results by using well known benchmark functions along with evaluation tools are revealed and discussed in Section 5. Conclusions of the study are presented in the last section of this paper.

## 2. Review of Genetic Algorithm Selection Process

There are no specific criteria or theoretical justification to choose an appropriate selection scheme for various problems. This can be an alarming situation due to the application of an inappropriate selection technique on numerical data which can lead to poor performance of the GA regarding reliability of the results. In this section, we will review the reproduction process of individuals and also will present the performance comparison regarding shortcomings and advantages of different selection schemes. Hence, there are several schemes for the selection of individuals from the population. So, for the purpose of conducting comparative performance evaluation studies, numerous GA selection techniques exist in the literature: roulette wheel selection/fitness proportional selection (RWS), linear rank selection (LRS), tournament selection (TS), stochastic remainder selection (SRS), etc.

Roulette wheel selection is another name of fitness proportional selection. This selection technique uses the proportion of the solutions which will affect the area in the wheel. The higher proportions will have a larger area in the wheel and vice versa. In RWS, the wheel will be partitioned according to the probability where the higher probability will have a bigger area and the lower probability will have a smaller area. In this selection technique, a circular wheel connected with a fixed pointer is used for choosing different individuals, which is on the border of the circular wheel [2]. The first individual is selected when the area of the circular wheel comes in front of the fixed pointer. The second individual is selected through the same procedure, and this procedure will be replicated till the selection of last individual. It is very obvious that the individual with highest fitness value will acquire the greater portion on circular wheel and will have a higher possibility of arriving in front of the wheel's fixed pointer when the wheel is spun. Therefore, the probability *p*_*i*_ of selecting individuals is directly proportional on its fitness value [19]:

(1) $$\begin{array}{c} p_{i}=\frac{f_{i}}{\sum_{j=1}^{W}f_{i}};\;i\in \left\{1,2,\ldots ,W\right\}, \end{array}$$

where *f*_i_ is a fitness value of *i*^th^ individual and *W* denotes the size of population.

RWS is a biased selection because the chance of the small area being selected is very low [2]. This selection scheme still has an advantage where the weaker solutions have a limited chance to be selected and may survive in the next generation [20, 21].

In the literature, there are some other selection techniques to overcome the above shortcomings. Hence, LRS is one of the most popular selection techniques, which is more beneficial to handle premature convergence issue as compared to RWS. This selection scheme is focused on rank-based selection procedure, which provides a better opportunity to weaker individuals in the context of uniform scaling. The chromosomes are selected with the probability *p*_*i*_ that is linearly proportional to the rank of chromosomes.

(2) $$\begin{array}{c} p_{i}=\frac{1}{W}\left(\varphi^{-}+\left(\varphi^{+}-\varphi^{-}\right)\frac{i-1}{W-1}\right);\;i\in \left\{1,2,\ldots ,W\right\}, \end{array}$$

where *i* is the rank of individual according to its fitness value and *W* is the size of population. Furthermore, *φ*^+^ and *φ*^−^ are parameters representing the best and worst selection of individuals linked with their ranks, respectively. For the estimation of the above function in equation (2), the constraints are *φ*^+^ = 2 − *φ*^−^and *φ*^−^ ≥ 0. The limitation of this scheme is slower convergence to optimal solution because difference between the best fitted chromosome and other chromosome is not significant due to closeness of values. So, LRS is more beneficial than other techniques due to standardized scaling procedure and also useful to overcome the problem of premature convergence [22].

TS is an extensively used selection technique in GAs. It is also applicable in most of the applied research problems. This selection scheme can be implemented competently and is amenable to parallelization [21]. The simplest form of TS is based on randomized selection of two individuals and conducting a competition to decide which chromosome will win and get selected for the mating pool, and then comparing it to a predetermined selection probability *p*_*i*_. Hence, the predetermined selection probability for individual *p*_*i*_ for (*t* − 1) tournament is given by

(3) $$\begin{array}{c} p_{i}=\frac{1}{W^{t}}\left({\left(W-i+1\right)}^{t}-{\left(W-i\right)}^{t}\right);\;i\in \left\{1,2,\ldots ,W\right\}, \end{array}$$

where *W* is defined as the population size and *t* is size of the tournament. For the binary tournament, *t* = 2, and for large tournament, *t* > 2. The probability of parameters provides a suitable procedure for adjusting the selection pressure. The TS can also be further extended to involve more than two individuals if desired [22].

The basic idea of the SRS technique is based on the deterministic sampling technique [20]. Each chromosome (individual) in the population has the selection probability based on its comparative fitness value. The SRS uses a concept of removing or copying the strings based on the values of the reproduction counts. The process is done by computing the reproduction count associated with each string. At first, the probability of selection *p*_*i*_,

(4) $$\begin{array}{c} p_{i}=\frac{f_{i}}{f_{\text{avg}}};\;i\in \left\{1,2,\ldots ,W\right\}, \end{array}$$

where *f*_i_ is a fitness value of *i*^th^ individual. Hence, the expected number of individuals in the mating pool is calculated as population size *W*:

(5) $$\begin{array}{c} e_{i}=p_{i}\;\times W. \end{array}$$

**Figure 2:**
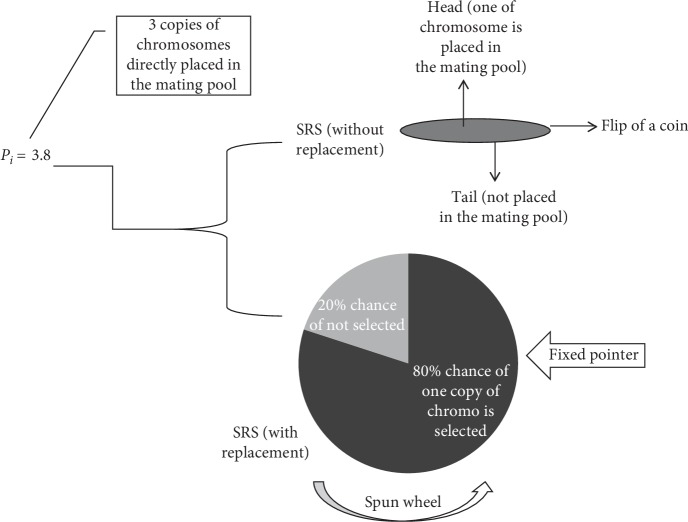
Stochastic remainder selection scheme.

Integer portion of *p*_*i*_ is used to choose as an individual deterministically and then uses RWS or flipping a coin to deal the remaining fractional portion and to fill the rest of portion in mating pool. For example, if the value of *p*_*i*_ = 3.8 as described in Figure 2, which means that three copies of chromosomes are directly placed in the mating pool because of integer portion, then the fractional portion of the parents are chosen stochastically.

There are two methods to deal with remainder portion of *p*_*i*_; the first is SRS with replacement and other is SRS without replacement. In SRS with replacement, the remainder part of *p*_*i*_ is used to size the portion of RWS process. The resultant probability is proportionate of fractional portion of its scaled value. This selection mechanism provides maximum opportunity of selecting best-fitted individuals of the population. In SRS without replacement technique, flipping a coin determines whether the fractional portion of scale value receives another copy or not.

## 3. Proposed Selection Scheme

### 3.1. Defining Problem

In the above context, most of the operators follow one extreme, i.e., exploitation or exploration. Therefore, for achieving the optimal solution, it is more beneficial to adjust selection pressure which maintained population diversity during the selection process. More illustratively, we considered RWS and LRS which are both extremes in selection of individuals [22]. Generally, LRS mainly focuses on maintaining population diversity (more technically known as exploration) by compromising selection pressure resulting delayed convergence and RWS emphasizes on selection pressure (known as exploitation) with shortcomings of premature convergence.

### 3.2. Proposed Scheme (Proportionate Selection)

To overcome the shortcomings of conventional selection schemes, we proposed a balanced selection approach associated with suitable tradeoff between exploitation and exploration, which basically decreases the effect of selection pressure and assure some genetic diversity within population. In other words, it will be a fine adjustment between selection pressure and loss of population diversity.

Here, the newly proposed selection scheme will be helpful in improving the search space through proportionate probabilistic approach. The initiation of probabilistic weights to individuals will definitely introduce greater diversity in the population, thus offering better solutions with sustainable convergence speed. Thus, the new selection scheme creates a sustainable adjustment between exploitation and exploration. Hence, a modified selection scheme is going to be proposed, named stairwise selection (SWS). Its objective is to overcome the disadvantages of other selection schemes by providing a comparatively better opportunity to the weak individuals for maintaining population diversity. This newly selection mechanism is designed in such a way that the resulting generation has a limited chance of deterioration.

The working phenomenon of SWS proceeds by assigning ranks to all individuals from worst to best criterion according to their fitness values. The ranked population of size *W* is given below:

(6) $$\begin{array}{c} 1+2+3+\cdots +\frac{W}{2}+\cdots +W. \end{array}$$

First, we divided the whole population into five equal portions as

(7) $$\begin{array}{c} \left[1+\cdots +\frac{W}{5}\right]+\left[\left(\frac{W}{5}+1\right)+\cdots +\frac{2W}{5}\right]+\left[\left(\frac{2W}{5}+1\right)+\cdots +\frac{3W}{5}\right]+\;\left[\left(\frac{3W}{5}+1\right)+\cdots +\frac{4W}{5}\right]+\;\left[\left(\frac{4W}{5}+1\right)+\cdots +N\right]. \end{array}$$

Hence, the selection probability of each individual “*i*” is according to the following function:

(8) $$\begin{array}{c} p_{i}=\left\{\begin{array}{cc} q_{1}\left(\frac{50i}{W\left(W+5\right)}\right); & 1\leq i\;\leq \;\frac{W}{5},\\ q_{2}\left(\frac{50i}{W\left(3W+5\right)}\right); & \frac{W}{5}<i\;\leq \;\frac{2W}{5},\\ q_{3}\left(\frac{10i}{W\left(W+1\right)}\right); & \frac{2W}{5}<i\;\leq \;\frac{3W}{5},\\ q_{4}\left(\frac{50i}{W\left(7W+5\right)}\right); & \frac{3W}{5}<i\;\leq \;\frac{4W}{5},\\ q_{5}\left(\frac{50i}{W\left(W+5\right)}\right); & \frac{4W}{5}<i\;\leq \;W, \end{array}\right. \end{array}$$

where *q*_1_ + *q*_2_ + *q*_3_ + *q*_4_ + *q*_5_ = 1 and the suitable probabilities weights are revealed in

(9) $$\begin{array}{c} p_{i}=\left\{\begin{array}{cc} \frac{i}{W\left(W+5\right)}; & 1\leq i\;\leq \;\frac{W}{5},\\ \;\frac{4.5i}{W\left(3W+5\right)}; & \frac{W}{5}<i\;\leq \;\frac{2W}{5},\\ \frac{9i}{W\left(5W+5\right)}; & \frac{2W}{5}<i\;\leq \;\frac{3W}{5},\\ \frac{15i}{W\left(7W+5\right)}; & \frac{3W}{5}<i\;\leq \;\frac{4W}{5},\\ \;\frac{20.5i}{W\left(9W+5\right)}; & \frac{4W}{5}<i\;\leq \;W. \end{array}\;\right. \end{array}$$

The pseudocode of SWS is given in Algorithm 1.

**Figure 3:**
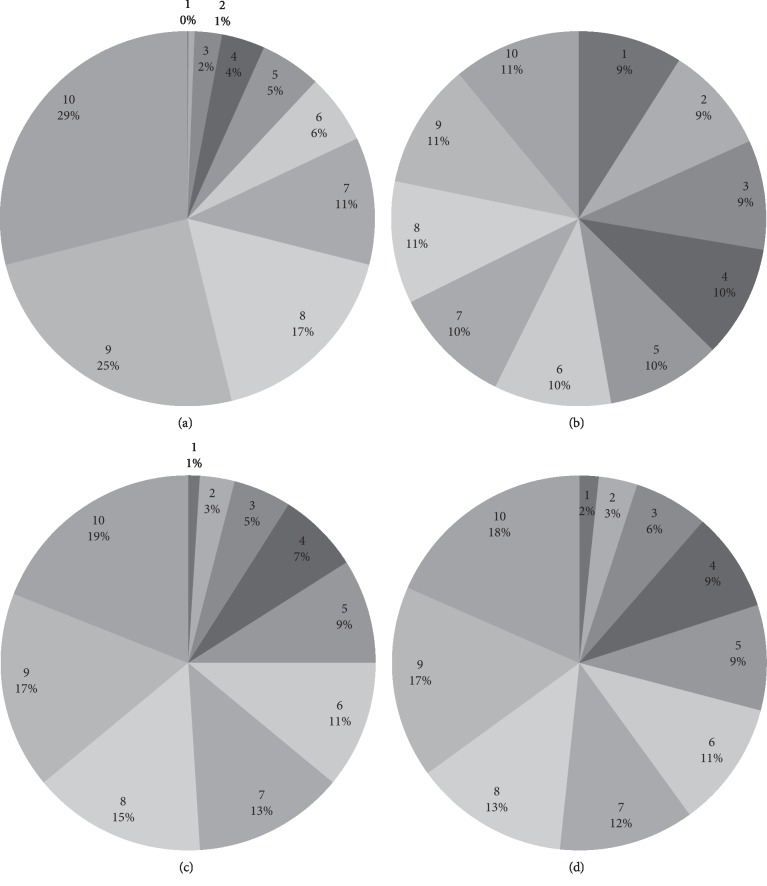
Comparative charts of selection scheme: (a) RWS, (b) LRS, (c) TS, and (d) SWS.

The performance of the GA is usually examined through the optimum value and number of generations required to get the optimum solution. For visual understanding and close comparison of different selection schemes, we considered a population of ten individuals.  Figure 3(a) shows that the individuals “1” to “3” have a limited chance to get selected because of the small portion in the roulette wheel instead of “7” to “10” with higher portion. Hence, current distribution of individuals in RWS increases selection pressure and reduce population diversity. Conversely, the distribution of LRS for individuals will delay the convergence due to uniform scaling.  Figure 3(c) shows that TS is giving more weight to individuals “1” to “3” as compared to RWS, which means that TS is somehow managing selection pressure and population diversity. Now, the newly proposed selection scheme, i.e., (SWS) has a better control over the above two extremes, i.e., selection pressure and population diversity. Because individuals “1” to “3” have a sufficient chance to be selected and “7” to “10” also have an adequate representation, there is an adequate balance between exploitation and exploration.

**Figure 4:**
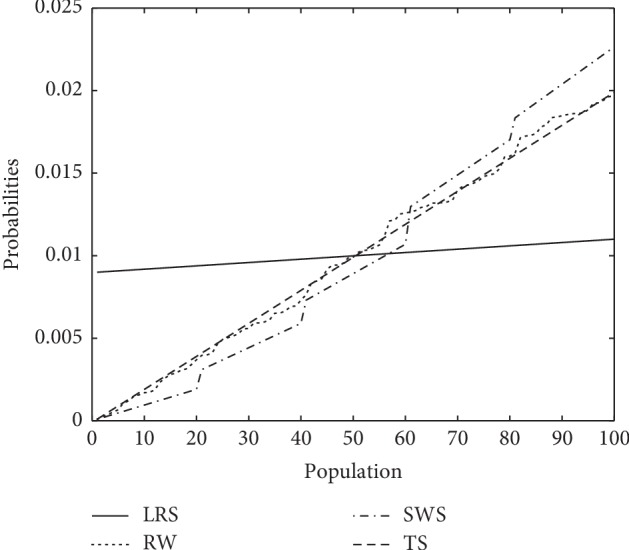
Comparative view of selection schemes.

For more realistic visual comparison, we considered a population of hundred individuals.  Figure 4 clearly visualizes that the graphical line of SWS occurs in between conventional selection schemes, which reflects that this novel selection scheme seems to have a better control over selection pressure, and it is more beneficial to maintain population diversity. In other words, it would be a perfect tradeoff between exploration and exploitation.

### 3.3. The Sampling Methodology

An efficient sampling procedure is required to select individuals for mating process through the mechanism of two-step selection. This sampling procedure fills the mating pool with copies of individuals of the given population, while respecting the selection probabilities *p*_*i*_, such that the observed and expected number of individuals are equal. Among the widely used sampling procedures, we commonly used the roulette wheel sampling technique (or Monte Carlo sampling) for evaluating the efficiency of the newly proposed SWS operator.

#### 3.3.1. Chi-Square Goodness-of-Fit Measure

*χ* ^2^ is used as a tool to measure the mean difference between observed and expected number of offspring. This measure was first time introduced by Schell and Wegenkittl [23] for average accuracy. Initially, there are *k* mutually exclusive classes as *C*={*C*_1_, *C*_2_, *C*_3_ …, *C*_*k*_}, where *C*_*j*_ ⊂ {1,  2,…,  *W*} and  ∪_*j*=1_^*k*^ *C*_*i*_={1,  2,….,*N*}. Let *ε*_*j*_=∑_*i*∈*C*_*j*__*e*_*i*_ denote the cumulative expectation and *O*_*j*_= ∑_*i*∈*C*_*j*__*o*_*i*_ represent the observed/actual copies of individuals in the mating pool followed by the sampling process. Preferably, the order of *ε*_*j*_ should be  *W*/*k*for 1 ≤ *j* ≤ *k*. So, on average, each class contains equal number of individuals, and there should be at least 10 number of classes to attain the required accuracy. Schell and Wegenkittl [23] suggested the Chi-square test as a measure to evaluate the efficiency of the sampling procedure as follows:

(10) $$\begin{array}{c} \chi =\overset{k}{\underset{j=i}{\sum }}\frac{{\left(\varepsilon_{j}-O_{j}\right)}^{2}}{\varepsilon_{j}}. \end{array}$$

In the context of the roulette wheel sampling scenario, the abovementioned constraint, i.e.,*ε*_*j*_  ≥ 10, *χ* should follow Chi-square distribution with *k* − 1 degree of freedom. This distribution is asymptotic of *χ* under multinomial distributed *o*_*i*_ when *W* ⟶ *∞*. According to the present research study, the concern-fixed parameters are the population size *W* = 100, number of classes = 10, and total number of tests *s* = 100.

The results in Table 1 reveal the probability distribution of SWS along with corresponding cumulative expectation, which are close to *W*/*k*=100/10. We used  *χ*^SW,*R*^ to evaluate the results of *χ*. In  *χ*^SW,*R*^, SW denotes the proposed operator that assigns selection probabilities to the individuals and *R* represents a technique of sampling algorithm. Mainly, this test is used to estimate the expectation and its variance. The population generated randomly with predefined specific individuals and used the probability distribution *R* to assign them probabilities for the process of selection followed by sampling procedure *R* is applied to obtain instance of *O*_*j*_ and *χ*^SW,*R*^, respectively. The sample mean and variance can be obtained through sequence (*χ*^SW,*R*^) with 1 ≤ *w* ≤ *s* as given below:

(11) $$\begin{array}{c} {\hat{e}}^{\left(\text{SW},R\right)}=\frac{1}{s}\overset{s}{\underset{W=1}{\sum }}\chi_{W}^{\text{SW},R},\\ {\hat{\sigma }}^{2\left(\text{SW},R\right)}=\frac{1}{s-1}\overset{s}{\underset{W=1}{\sum }}{\left(\chi_{W}^{\text{SW},R}-\;{\hat{e}}^{\text{SW},R}\right)}^{2}. \end{array}$$

For the purpose of evaluation, this technique is compared with theoretical distribution *χ*_*k*−1_^2^ at 99% confidence level. The mean and variance of *χ*^2^ distribution are *k* − 1 = 9 and 2(*k* − 1) = 18 for 10 classes. Hence, the corresponding estimates of $\hat{e}$ and ${\hat{\sigma }}^{2}$ are 9.1025 and 19.8583, respectively. The above estimates are almost similar and comparatively more accurate in terms of symbolic representation between assigning probabilities to the individuals and the number of copies related to their respective probabilities coming in the mating pool. The simulated results authenticate the overall performance of the sampling procedure with respect to probability distribution of SWS. Hence, the roulette wheel sampling technique provides the empirical distribution function that cannot be significantly different from theoretical distribution *χ*_*k*−1_^2^ regarding $\hat{e}$ and ${\hat{\sigma }}^{2}$ estimates.

## 4. Benchmark Functions

There is not a rule of thumb for the evaluating the performance of the GA by choosing an appropriate optimization function. Therefore, the performance of the algorithm is based on the nature of the problem regarding variation rate in objective function, the number of local optima, etc. [24]. A multimodal function has at least two local optima. The efficient search procedure must be proficient of eliminating the region around local optimum in context of the search for global optima. The scenario becomes more complex in situation of random distribution of local optima in the search space.

The dimensionality of the search space is another significant factor which makes the problem more complicated. A comprehensive study regarding dimensionality problem and its characteristics was carried out by Friedman [25]. During the search process, value regarding global optimum needs to be obtained efficiently. Hence, the areas close to local minima must be avoided as much as possible. If the local optima are randomly distributed in the search area, then it is considered to be a most difficult problem. The optimization process focuses on obtaining the global optimum point; consequently, the regions nearby local optima should be circumvented because the optimization process might be stuck at local optima and then local optima are considered to be as global optima. To evaluate the performance and sustainability of the proposed selection operators, we used ten unimodal, multimodal, separable or nonseparable, convex, and continuous benchmark functions.  Table 2 presents the list of benchmark functions [16, 26–42] utilized to appraise the efficiency of the suggested evolutionary methods. Hence, the benchmark function's name, limit, properties, and fitness function are presented in Table 2. These benchmark factions have varying complexities that are most commonly applied in many comparative studies. The necessary details regarding these benchmarks are given below:

## 5. Computational Results and Discussions

### 5.1. Experimental Setup

In this section, we focused on the experimental results of four conventional and one proposed GA selection schemes. The overall efficiency of these selection schemes can be influenced by the use of fixed parameters with additional experimental conditions. Hence, the suitable values for fixed parameters such as population size, crossover and mutation probability, number of generation, and scaling function.  Table 3 shows the value of fixed parameters that are used for optimization problems. The performance of these selection schemes is evaluated on ten benchmark functions using MATLAB version R2015a. The simulated results of these runs are obtained in terms of mean and standard deviation (S.D). An independent *t*-test is also executed to examine the significant difference between different selection schemes. The *p* value along with mean and S.D of thirty runs are reported in subsequent tables. The sign of “^*∗*^” indicates the significant difference with the proposed technique and “a” defines the significance difference with reference technique.

### 5.2. Experimental Results

In this experimental study, the optimum values regarding GA were obtained through screening experimentation and trial run. The algorithms were executed thirty times, and the mean value and standard deviation are taken as final results. All experiments are terminated in this study when number of generations achieved the maximum numbers of generation.

The basic objective of this study is to make a comparison between different conventional selection schemes with the proposed one in the context of optimal solution by using benchmark functions. The overall statistical results of Table 4 clearly show that SWS obtained a minimum mean value and low S.D compared to other selection techniques from 10 to 100 dimensions. But there is a nonsignificant difference between SWS and TS at some benchmark functions. As we can notice for Axis Parallel Hyper Ellipsoid function, when dimensions of the study increase from 10 to 100, the average rate of change is in between 706 and 3052 because of function complexity. Hence, the minimum average rate of change is 706 in SWS and maximum is 3052 under RW at lower dimensions. The *p* value of *t*-tests further reduced with increase in the dimensions of experiment that actually tends toward significance of the results. About Colville function, SWS is the best-performing selection technique with the mean value of 1.39 at 10 dimensions with highly significant differences. When we increase the dimensions up to 100, the optimum value increases up to 5940 in the Colville function. Hence, the average rate of change is much high due to complexity. According to Table 4, the results of Ellipsoidal family function reveal that the proposed selection scheme (SWS) is the best-performing approach with minimum mean value of 0.0000 at lower dimensions, but at higher dimensions, the average rate of change is 187286 which is at the higher side. Another unimodal function is Rosenbrock; its statistical results about SWS are close to the theoretical optimum value which means that the proposed selection technique is efficiently handle complex problems at higher dimensions. The average rate of change in the Schaffer function is considerably low which shows that SWS efficiently performs at higher dimensions. The optimum value of SWS is ranging from 4.14 to 45.61 in the Schaffer function for 10–100 dimensions.

According to the results of the Beale function, Table 5 shows that the optimum value is obtained through TS. Moreover, SWS has significant difference with LRS but not with RWS, TS, and SRS at lower dimensions. When we increase the dimension, *p* value will also reduce from 0.9807 to 0.0000, and the average rate of change of TS is 583 which is considered close to the theoretical optimum value as compared to other selection techniques including SWS.

The SWS also achieves the minimum average rate on the Bohachevsky function, i.e., 98. Furthermore, the average rate of change is 84 for SWS which is the lowest in all other schemes from low to high dimensions. Moreover, the results of Bohachevsky benchmark function in Table 5 reveal that SWS distinctly performs better than all other selection schemes in terms of least empirical values. Moreover, by increasing the dimensions of experiment, SWS significantly differs at higher dimensions and show nonsignificance difference at lower dimensions with TS and RS.

According to the results in Table 5, SWS is considerably close to the theoretical optimum value under Drop-wave and Egg-holder benchmark functions, but the average rate of change in Egg-holder function is much higher when we increase the dimensions as compare to Drop-wave function. Hence, SWS efficiently handles selection pressure and makes improvements in population diversity at broader dimensions due to minimum average rate of change. In the Schwefel multimodal function, the empirical value is between −2898 and −11872 from low to high dimensions, which are quite high with reference to the theoretical optimum value due to the complexity of function. Overall statistical results of multimodal functions show that SWS outperforms than other selection techniques along with highly significant difference.

In the context of above discussion, it is demonstrated the substantial amount of effectiveness of the newly proposed selection technique over the standard GA techniques. Additionally, SWS selection technique ensured a broader and comprehensive search and avoided premature convergence to the optimum solutions in unimodal and multimodal benchmark functions. The newly proposed technique efficiently handles the problem of selection pressure and extends the diversity by intensifying the scope of the search process. This scheme is also reducing the possibility of less favorable solutions at higher as well as lower dimensions. In addition, the proportionate selection strategy ensures that best solutions are always carried forward to the next generation. In fact, SWS enhances the exploration of future generations and reduces the chance of premature convergence at local minima.

### 5.3. Overall Performance

The empirical results of conventional selection schemes (RWS, TS, LRS, and SRS) along with proposed SWS are evaluated on ten benchmark functions. The statistical results of Table 6 reveal that SWS outperforms in almost all benchmark functions regarding robustness, stability, and effectiveness of the solutions.

TS is the second best selection scheme because its optimum values are considerably close to SWS and sometimes have nonsignificant difference between these two. SWS equally efficient for unimodal and multimodal functions but the average rate of change is comparatively high in multimodal functions. Furthermore, SWS also performs efficiently when increasing the dimensions of experiment from 10 to 100 and also establish a suitable adjustment between exploitation and exploration. The influence of results in Table 6 confirms that SWS has a firm grip on controlling selection pressure and population diversity.

### 5.4. Performance Index (PI)

After descriptively evaluating the performance of stairwise selection operator with others, our next goal is to make a comparison between GAs' selection schemes based on relative performance index (PI) defined by Bharti [43]. This performance index was specifically used to analyze the behavior of some controlled stochastic search techniques. The PI is a widely used mechanism for comparing population-based heuristic algorithms [44, 45]. The PI can be mathematically derived in following way:

(12) $$\begin{array}{c} \text{PI}=\frac{1}{W_{\text{p}}}\overset{W_{\text{p}}}{\underset{i=1}{\sum }}\left(\theta_{1}\beta_{1}^{i}+\theta_{2}\beta_{2}^{i}+\theta_{3}\beta_{3}^{i}\right), \end{array}$$

where

(13) $$\begin{array}{c} \beta_{1}^{i}=\frac{M^{i}}{{\text{LM}}^{i}},\\ \beta_{2}^{i}=\frac{S^{i}}{{\text{LS}}^{i}},\\ \beta_{1}^{i}=\frac{{\text{MAE}}^{i}}{{\text{LMAE}}^{i}},\\ \text{for }i=1,2,\ldots ,\;W, \end{array}$$

where *M*^*i*^ = mean value of objective function for *i*^th^ optimization problem, *LM*^*i*^ = least mean value of objective function obtained by all algorithms for *i*^th^ optimization problem, *S*^*i*^ = standard deviation of objective function for *i*^th^ optimization problem, *LS*^*i*^ = least standard deviation value of objective function obtained by all algorithms for *i*^th^ optimization problem, MAE^*i*^ = the value of mean absolute error of objective function for *i*^th^ optimization problem, LMAE^*i*^ = least mean absolute error value of objective function obtained by all algorithms for *i*^th^ optimization problem, *W*_p_ = the total population to be analyzed.

*θ*_1_, *θ*_2_, and *θ*_3_ (*θ*_1_ + *θ*_2_ + *θ*_3_ = 1 and 0 *≤* *θ*_1_, *θ*_2_, *θ*_3_ ≤ 1) are weights assigned to three statistics that were considered, respectively.

In the context of the above definition, it is revealed that PI is a function of *θ*_1_, *θ*_2_, and *θ*_3_, respectively. Since *θ*_1_ + *θ*_2_ + *θ*_3_ = 1, one of *θ*_*i*_, *i* = 1, 2, 3 could be eliminated to reduce the number of dependent variables from the expression of PI (equation (12)). However, it is still difficult to graphically examine the behavior of all GAs' selection techniques due to overlapping of the surface plot of PI. So, we adopt the modified mechanism is the subsequent section by assigning same weights to any two terms in PI (equation (12)). Hence, the PI becomes a function of single variable. The resultant cases are given below:

(14) $$\begin{array}{c} \left(\text{case }1\right)\;\theta_{1}=wt,\;\theta_{2}=\theta_{3}=\frac{1-wt}{2}\;,\;\text{where }0\leq wt\leq 1,\\ \left(\text{case }2\right)\;\theta_{2}=wt,\;\theta_{1}=\theta_{3}=\frac{1-wt}{2}\;,\;\text{where }0\leq wt\leq 1,\\ \left(\text{case }3\right)\;\theta_{3}=wt,\;\theta_{1}=\theta_{2}=\frac{1-wt}{2},\;\text{ where }0\leq wt\leq 1. \end{array}$$

**Figure 5:**
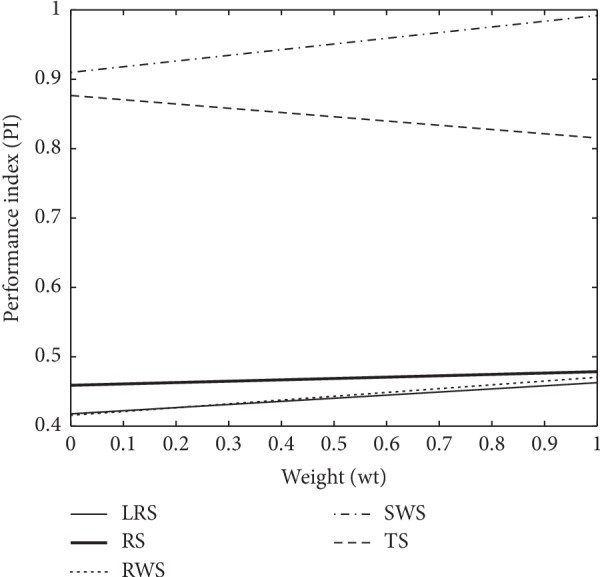
The working strategy of GA selection operators with proposed SWS for case 1.

**Figure 6:**
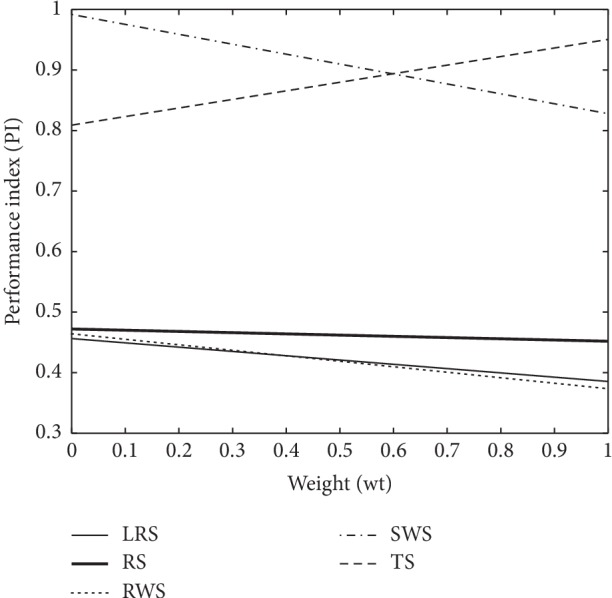
The working strategy of GA selection operators with proposed SWS for case 2.

**Figure 7:**
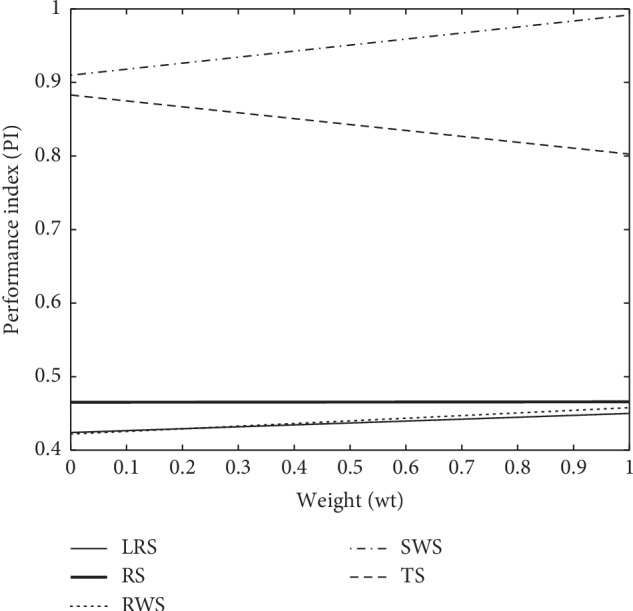
The working strategy of GA selection operators with proposed SWS for case 3.

The graphical representation for cases (1–3) in Figures 5–7 reveal that the horizontal axis define weights (wt) and performance index (PI) scaled on the vertical axis. The PI of proposed SWS is superimposed in Figures 5 and 7 as compared to other selection schemes which show a substantial enhancement towards perfection. Moreover, SWS shows considerable improvement at lower weights in terms of PI in Figure 6. More specifically, the graphical representation of PI endorses the improved performance of SWS.

## 6. Conclusions

In current study, we focused on the relative performance among various selection techniques to obtain the optimal solution for given test problems. A set of selection techniques including roulette wheel selection (RWS), linear rank selection (LRS), tournament selection (TS), stochastic remainder selection (SRS), and stairwise selection (SWS) were considered, and their performance was evaluated through ten well-known benchmark functions with 10 to 100 dimensions. These benchmark functions cover various characteristics including convex, separable, nonseparable, unimodal, and multimodal. Additionally, the results of Chi-square goodness of fit test show improvements regarding proposed selection technique, and there is also an insignificant difference between expected and actual number of offsprings. The statistical results of this study also show that the proposed selection technique (SWS) performed best in nine out of ten benchmark functions because of proportionate selection methodology. Furthermore, the simulated results reveal that the performance of SWS is significantly improved for unimodal and multimodal benchmark functions. When increasing the dimensions of experiments, SWS also performed efficiently under complex circumstances of dimensionality. The variability of results reveals that the proposed scheme has a better control over selection pressure and loss of population diversity. Therefore, SWS found a suitable adjustment between exploitation and exploration due to split ranked ideology. According to the results, TS is the second best selection technique after SWS, and sometimes there is insignificance difference between these two. Finally, the numerical outcomes of proposed technique are very close to theoretical optimum value which is an evidence of the best-performing selection technique with authentication of performance index (PI).

## Figures and Tables

**Algorithm 1:**
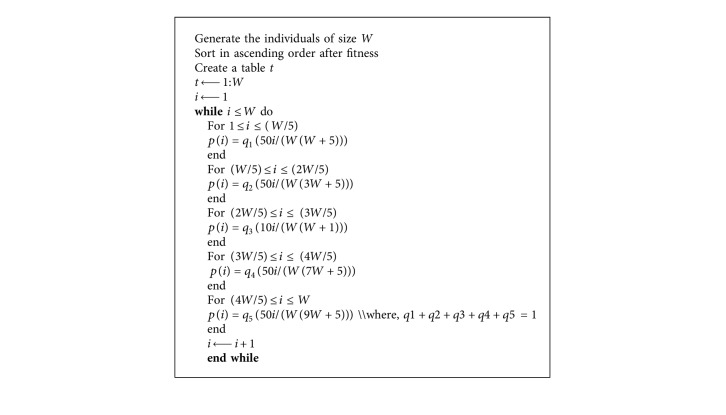
The pseudocode of stairwise selection scheme.

**Table 1:** Classes of *C*_*j*_ and overall expectation *ε*_*j*_ for SWS.

*j*	*C*_*j*_	*ε*_*j*_
1	1–28	9.8196
2	29–40	10.1803
3	41–51	10.0198
4	52–60	9.9802
5	61–69	10.3723
6	70–77	10.4255
7	78–84	10.5833
8	85–90	10.1519
9	91–95	8.9917
10	96–100	9.4751

**Table 2:** Detail of benchmark functions for comparison.

Benchmark	Fitness function	Search limits	Optimum value	Properties
Axis parallel ellipsoid	*f*(*x*)=∑_*i*=1_^*D*^*ix*_*i*_^2^	[−5.12, 5.12]	0	Continues, convex, unimodal
Beale	*f*(*x*)=∑_*i*=1_^*D*^(1.5 − *x*_*i*_+*x*_*i*_*x*_*i*+1_)^2^+(2.25 − *x*_*i*_+*x*_*i*_*x*_*i*+1_^2^)^2^+(2.625 − *x*_*i*_+*x*_*i*_*x*_*i*+1_^3^)^2^	[−4.5, 4.5]	0	Multimodal, nonseparable
Continues				
Bohachevsky	*f*(*x*)=∑_*i*=1_^*D*^*x*_*i*_^2^+2*x*_*i*+1_^2^ − 0.3 cos(3*πx*_*i*_) − 0.4 cos(4*πx*_*i*+1_)+0.7	[−100, 100]	0	Multimodal, nonseparable
Colville	*f*(*x*)=∑_*i*=1_^*D*^100(*x*_*i*_^2^ − *x*_*i*+1_)^2^+(*x*_*i*_ − 1)^2^+(1 − *x*_*i*+2_)^2^+90(*x*_*i*+3_ − *x*_*i*+2_^2^)^2^+10.1(*x*_*i*+1_ − 1)^2^+(*x*_*i*+3_ − 1)^2^+19.8(*x*_*i*+1_ − 1)(*x*_*i*+3_ − 1)	[−10, 10]	0	Unimodal, nonseparable
Drop-wave	$f\left(x\right)=\sum_{i=1}^{D}\left(1+\cos\left(12\sqrt{x_{i}^{2}+x_{i+1}^{2}}\right)\right)/\left(0.5\left(x_{i}^{2}+x_{i+1}^{2}\right)+2\right)$	[−5.12, 5.12]	−1	Multimodal, nonseparable
Egg-holder	$f\left(x\right)=\Sigma_{i=1}^{D}-\left(x_{i}+47\right)\sin\;{\left(\sqrt{\left|x_{i+1}+\left(x_{i}/2\right)+47\right|}-x_{i}\sin\left(\;\right.\sqrt{\left|x_{i}+x_{i+1}+47\right|}\right)}^{1}$	[−5.12, 5.12]	−959.6407	Nonconvex, multimodal
Ellipsoidal	*f*(*x*)=∑_*i*=1_^*D*^(*x*_*i*_ − *i*)^2^	[−*n*, *n*]	0	Unimodal
Rosenbrock	*f*(*x*)=∑_*i*=1_^*D*^(100(*x*_*i*_^2^ − *x*_*i*+1_)^2^+(1 − *x*_*i*_)^2^)	[−2.048, 2.048]	0	Unimodal, nonseparable
Schaffer	*f*(*x*)=∑_*i*=1_^*D*^0.5+((sin (*x*_*i*_^2^+*x*_*i*+1_^2^)^2^ − 0.5)/(1+0.001(*x*_*i*_^2^+*x*_*i*+1_^2^)^2^))	[−100, 100]	0	Unimodal, nonseparable
Schwefel	$f\left(x\right)=\sum_{i=1}^{D}x_{i}\sin\left(\sqrt{\left|x_{i}\right|}\right)$)	[−500, 500]	0	Multimodal, nonseparable

**Table 3:** Specific parameters for GAs' working strategy.

Parameter	Value
Population size	100
Fitness scaling	Proportional/rank
Elite count	0.05
Crossover fraction	0.8
Crossover operator	Two point
Migration fraction	0.2
Generations	200
Function tolerance	1.*E* − 06
Mutation function	Gaussian

**Table 4:** Statistical results of optimum values for different selection schemes using unimodal benchmark functions.

Benchmark	Selection schemes						
	Dimension	Statistics	RW	TS	RS	LRS	SWS
Axis parallel hyper ellipsoid	10	Mean	5.0835*E* − 05	3.2320*E* − 07	1.5786*E* − 05	3.3563*E* − 05	2.9418*E* − 07
		S.D	7.9800*E* − 05	2.5215*E* − 07	3.5962*E* − 05	5.8134*E* − 05	2.2951*E* − 07
		*t*-test	0.00099	0.64287	0.02170	0.00619	
	50	Mean	3.1488*E* + 02	1.9369*E* + 01	3.0683*E* + 02	3.2088*E* + 02	1.7630*E* + 01
		S.D	1.8473*E* + 02	1.0019*E* + 01	1.9457*E* + 02	1.9967*E* + 02	9.1199*E* + 00
		*t*-test	0.00000	0.48486	0.00000	0.00000	
	100	Mean	3.0516*E* + 03	7.7562*E* + 02	2.8707*E* + 03	3.1637*E* + 03	7.0599*E* + 02
		S.D	8.2320*E* + 02	2.0261*E* + 02	7.9006*E* + 02	1.0092*E* + 03	1.8442*E* + 02
		*t*-test	0.00000	0.16921	0.00000	0.00000	

Colville	10	Mean	14.1036	1.4075	5.2867	10.0411	1.3926
		S.D	64.1057	1.9450	24.4547	44.6261	1.9245
		*t*-test	0.2822	0.9763	0.3882	0.0001	
	50	Mean	2465.5693	613.2918	2293.8499	2380.0555	606.8044
		S.D	2717.5439	287.6108	1873.1595	2295.6976	284.5685
		*t*-test	0.0004	0.9303	0.0000	0.0000	
	100	Mean	19237.7118	6003.8649	20567.1507	19902.7771	5940.3560
		S.D	6243.5664	1519.1380	8549.9094	7397.0838	1503.0685
		*t*-test	0.0000	0.8713	0.0000	0.0000	

Ellipsoidal	10	Mean	5.8835*E* − 06	3.6933*E* − 07	2.1295*E* − 05	5.1129*E* − 05	3.3639*E* − 07
		S.D	8.9408*E* − 06	4.7645*E* − 07	8.7336*E* − 05	5.4186*E* − 05	4.3396*E* − 07
		*t*-test	0.00125	0.78053	0.19389	0.00477	
	50	Mean	2408.7101	716.6584	2228.5647	2423.9663	652.7483
		S.D	941.5905	392.6978	784.5990	956.8467	357.6778
		*t*-test	0.0000	0.5125	0.0000	0.0000	
	100	Mean	87681.5841	34427.9838	83676.4636	87696.8404	31357.7650
		S.D	18184.3183	9613.1629	14342.6643	18199.5746	8755.8803
		*t*-test	0.0000	0.2010	0.0000	0.0000	

Rosenbrook	10	Mean	6.9873	7.0153	5.9280	8.2780	6.4416
		S.D	3.6870	1.8204	3.0867	5.2072	1.6715
		*t*-test	0.0000	0.0000	0.0000	0.0000	
	50	Mean	444.0538	263.3415	348.7025	443.6579	241.8055
		S.D	159.6747	47.2798	110.5376	182.3859	43.4132
		*t*-test	0.0000	0.0712	0.0000	0.0000	
	100	Mean	3491.3158	1296.4477	2971.5222	3461.4764	1190.4242
		S.D	1123.5970	230.0574	653.2587	1118.4853	211.2433
		*t*-test	0.0000	0.0681	0.0000	0.0000	

Schaffer	10	Mean	4.5650	4.5645	4.5653	4.5694	4.1455
		S.D	0.0089	0.0084	0.0076	0.0103	0.0076
		*t*-test	0.0000	0.0000	0.0000	0.0000	
	50	Mean	25.5110	25.2177	25.1508	25.5793	22.2471
		S.D	0.1419	0.0708	1.9232	0.1418	0.0643
		*t*-test	0.0000	0.0000	0.0000	0.0000	
	100	Mean	52.6225	51.7076	52.5572	52.9025	45.6164
		S.D	0.2535	0.2024	0.3154	0.3309	0.1838
		*t*-test	0.0000	0.0000	0.0000	0.0000	

**Table 5:** Statistical results of optimum values for different selection schemes using multimodal benchmark functions.

Selection schemes							
Benchmark	Dimension	Statistics	RW	TS	RS	LRS	SWS
Beale	10	Mean	2.5691*E* + 01	2.3543*E* + 01	2.5206*E* + 01	3.0214*E* + 01	2.5657*E* + 01
		S.D	5.5649*E* + 00	4.7657*E* + 00	5.4125*E* + 00	5.4887*E* + 00	5.1936*E* + 00
		*t*-test	0.98075	0.10591	0.74302	0.01616	
	50	Mean	3.2013*E* + 02	2.1626*E* + 02	2.8608*E* + 02	3.2635*E* + 02	2.3568*E* + 02
		S.D	6.3963*E* + 01	2.3247*E* + 01	3.9857*E* + 01	5.1910*E* + 01	2.5334*E* + 01
		*t*-test	0.00000	0.00305	0.00000	0.00000	
	100	Mean	9.9730*E* + 02	6.0705*E* + 02	9.7269*E* + 02	1.0248*E* + 03	6.6155*E* + 02
		S.D	1.9613*E* + 02	3.9781*E* + 01	1.5325*E* + 02	1.7469*E* + 02	4.3353*E* + 01
		*t*-test	0.00000	0.00000	0.00000	0.00000	

Bohachevsky	10	Mean	5.5629*E* − 01	5.5020*E* − 07	2.2209*E* − 01	3.8920*E* − 01	5.0851*E* − 07
		S.D	1.1163*E* + 00	3.9736*E* − 07	4.9787*E* − 01	8.0708*E* − 01	3.6725*E* − 07
		*t*-test	0.00838	0.67454	0.01762	0.00000	
	50	Mean	88.2869	19.2995	92.1134	93.8072	17.8370
		S.D	29.3399	3.6071	26.7442	31.6492	3.3338
		*t*-test	0.0000	0.1083	0.0000	0.0000	
	100	Mean	251.3332	105.8799	267.6916	274.2843	97.8563
		S.D	41.7324	14.7719	49.6698	45.7011	13.6525
		*t*-test	0.0000	0.0330	0.0000	0.0000	

Drop-wave	10	Mean	−8.4413	−8.3885	−8.4403	−8.4322	−4.4669
		S.D	0.1928	0.2039	0.1480	0.3682	0.5363
		*t*-test	0.0000	0.0000	0.0000	0.0000	
	50	Mean	−36.9639	−37.6504	−37.5574	−37.2521	−17.9598
		S.D	1.7057	1.4806	1.8322	1.9668	1.9511
		*t*-test	0.0000	0.0000	0.0000	0.0000	
	100	Mean	−65.0661	−67.0406	−66.3824	−65.7157	−35.1363
		S.D	6.5688	2.7298	2.7023	4.8334	4.9423
		*t*-test	0.0000	0.0000	0.0000	0.0000	

Egg-holder	10	Mean	−608.5168	−608.5186	−608.5177	−608.5087	−413.0947
		S.D	0.0021	0.0008	0.0017	0.0020	21.3483
		*t*-test	0.0000	0.0000	0.0000	0.0000	
	50	Mean	−3318.3868	−3320.1156	−3318.4740	−3317.6446	−1875.4208
		S.D	1.5810	0.5672	1.2800	1.4306	200.0058
		*t*-test	0.0000	0.0000	0.0000	0.0000	
	100	Mean	−6672.6025	−6694.8362	−6678.1726	−6674.6017	−3541.7138
		S.D	9.4819	4.4452	8.4881	8.9851	251.8305
		*t*-test	0.0000	0.0000	0.0000	0.0000	

Schwefel	10	Mean	−4020.3795	−4062.8059	−4083.1594	−4065.7950	−2898.0973
		S.D	137.7577	114.5713	125.9619	117.8343	249.1698
		*t*-test	0.0000	0.0000	0.0000	0.0000	
	50	Mean	−15391.3838	−14734.6805	−15825.5138	−15622.4744	−8337.1457
		S.D	849.1259	822.2196	766.8707	793.9728	785.8473
		*t*-test	0.0000	0.0000	0.0000	0.0000	
	100	Mean	−24086.2209	−23310.1510	−25169.1100	−24641.6910	−11872.9339
		S.D	1712.0336	1468.7940	1456.0778	1570.0301	1476.5720
		*t*-test	0.0000	0.0000	0.0000	0.0000	

**Table 6:** Cumulative results of best selection techniques.

Functions	Dimensions		
	10	50	100
Axis parallel hyper ellipsoid	2.9418*E* − 07 (SWS)	1.7630*E* + 01 (SWS)	7.0599*E* + 02 (SWS)
Beale	23.5433 (TS)	216.2626 (TS)	607.0514 (TS)
Bohachevsky	5.0851*E* − 07 (SWS)	17.8370 (SWS)	97.8563 (SWS)
Colville	1.3926 (SWS)	606.8044 (SWS)	5940.3560 (SWS)
Drop-wave	−4.4669 (SWS)	−17.9598 (SWS)	−35.1363 (SWS)
Egg-holder	−413.0947 (SWS)	−1875.4208 (SWS)	−3541.7138 (SWS)
Ellipsoidal	3.3639*E* − 07 (SWS)	652.7483 (SWS)	31357.7650 (SWS)
Rosenbrook	6.4416 (SWS)	241.8055 (SWS)	1190.4242 (SWS)
Schaffer	4.1455 (SWS)	22.2471 (SWS)	45.6164 (SWS)
Schwefel	−2898.0973 (SWS)	−8337.1457 (SWS)	−11872.9339 (SWS)

## Data Availability

The data used to support the findings of this manuscript are taken from the website (https://www.sfu.ca/ssurjano/optimization.html).
